# Sinus of Valsalva Fistula to the Right Ventricle along with Coronary Artery Fistula to the Pulmonary Artery in a Young Native American Female

**DOI:** 10.1155/2013/674608

**Published:** 2013-11-19

**Authors:** Sarika Desai, Erica Flores, Akil Loli, Peter Maki

**Affiliations:** ^1^Banner Good Samaritan Medical Center, USA; ^2^Cardiology Fellowship Office, Banner Good Samaritan Medical Center, 1111 East McDowell Road, Phoenix, AZ 85006, USA; ^3^Department of Cardiology, Biltmore Cardiology/Banner Good Samaritan Medical Center, USA

## Abstract

Sinus of Valsalva aneurysm is a rare condition and associated with a high rate of mortality if rupture occurs. The aneurysms are rarely diagnosed until rupture occurs. This case describes a young Native American female whose only symptom was intermittent chest pain prior to the detection of the aneurysm along with a small ventricular septal defect. The patient was also found to have a coexisting coronary artery fistula, and it is rare phenomenon to have these coexisting anomalies. The anomalies were demonstrated on both cardiac computed tomography and coronary angiography. The patient underwent surgical closure of both anomalies, which is the recommended treatment to avoid future complications.

## 1. Introduction

Sinus of Valsalva fistulas was first described in 1839. The incidence of sinus of Valsalva fistulas ranges considerably in previously conducted studies form 0.14% to 0.96% [1–3]. In one study by Takach and colleagues, the most common site of origin was the right coronary sinus with rupture into the right ventricle. Ventricular septal defects were an associated finding in 11.6% of cases, which was a finding in our patient. The absolute indications for surgical intervention in a ruptured fistula include right ventricular outflow obstruction, infection, arrhythmias, or coronary artery obstruction [4]. However, the literature is less definitive regarding the optimal management for asymptomatic, nonruptured fistulas. 

## 2. Case Presentation

A 32-year-old Native American female was referred to cardiology for a newly diagnosed heart murmur and a history of previously diagnosed congenital heart disease. The patient reported atypical chest pain intermittently upon awakening but denies any palpitations or dyspnea. The patient had been diagnosed with a cardiac abnormality and murmur at birth, but she believed the murmur and abnormality had resolved. The patient was previously diagnosed with mild developmental delay, hyperthyroidism, and asthma. She lives with her parents and denies any alcohol or drug use. There was no family history of congenital heart disease. Clinical examination revealed a 3/6 systolic murmur at the left lower sternal border radiating to the apex. A continuous, high-pitched, and diastolic murmur was also were noted at the left sternal border. 

**Figure 1:**
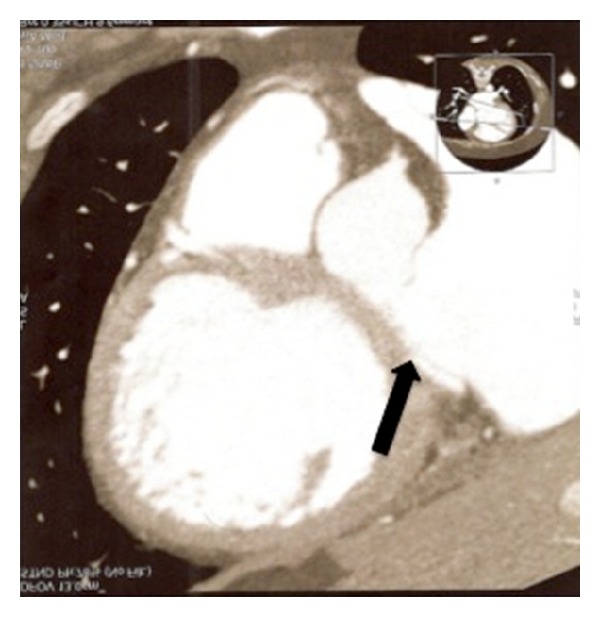
Cardiac CT demonstrating aortic right ventricular fistula.

**Figure 2:**
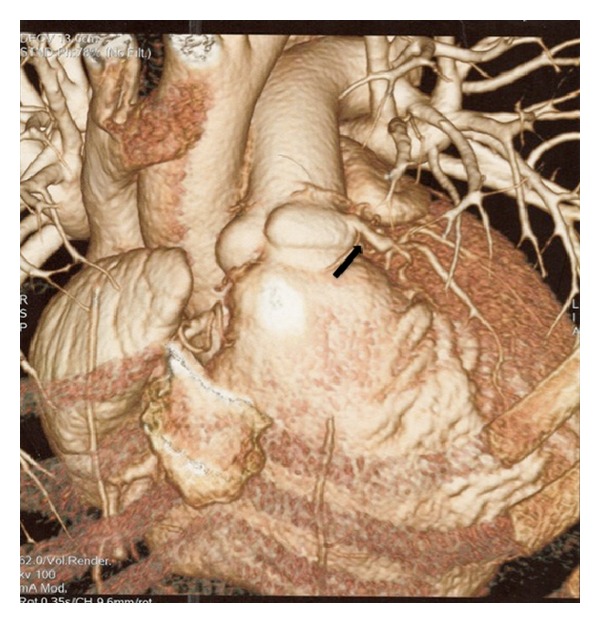
Cardiac CT demonstrating aortic right ventricular fistula.

 The transthoracic echocardiogram showed an intracardiac shunt from the right coronary sinus of Valsalva to the right atrium creating inward movement towards the right atrium of the tricuspid valve during systole. The echocardiogram also revealed mild to moderate tricuspid regurgitation, right atrial enlargement, and right and left ventricles with normal size and function. Cardiac computed tomography angiography was obtained for better anatomical delineation of the abnormality, which showed presumably no evidence of coronary artery obstruction and a small ventricular septal defect in the mid-septum. The sinuses of Valsalva were found to be prominent with communication between the right coronary sinus at its base and the right ventricle (Figures 1 and 2). The computed tomography of the chest was unremarkable.

**Figure 3:**
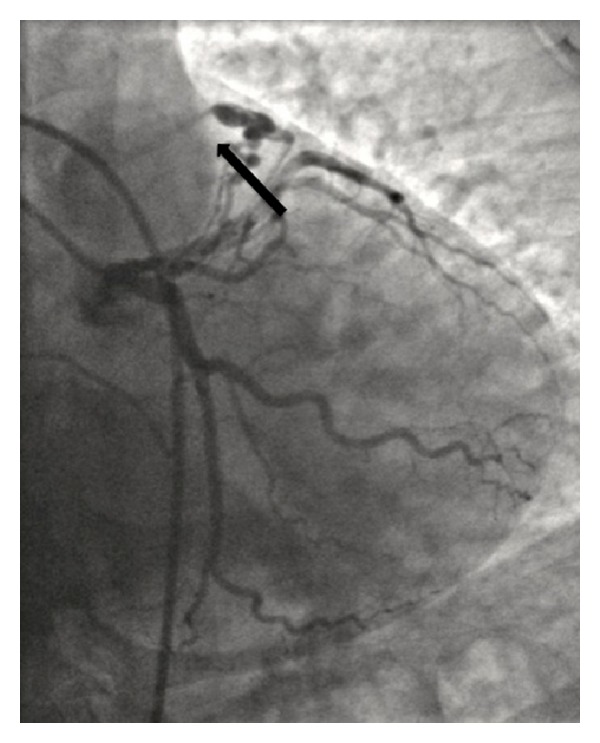
Coronary angiography demonstrating fistula between the proximal circumflex artery and pulmonary artery.

**Figure 4:**
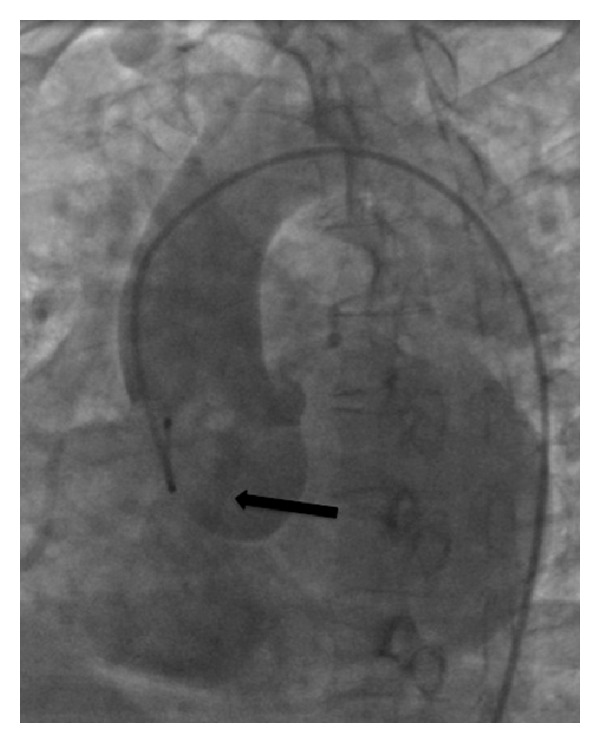
Aortic angiography demonstrating aortic right ventricular fistula.

The patient underwent a right and left cardiac catheterization with aortography. The coronary angiography demonstrated a fistula between the proximal circumflex artery and the pulmonary artery (PA) (Figure 3). Left ventriculography was normal. Aortic angiography showed passage of contrast between the aortic root and right heart chambers (Figure 4). There was evidence of a large left to right shunt with a Qp : Qs ratio of 2.7 : 1.

After testing was completed, the patient was ultimately found to have a sinus of Valsalva aneurysm of the right coronary sinus, an aorto-right ventricular fistula, a proximal circumflex artery to pulmonary artery fistula, and a small ventricular septal defect. She was sent for consultation with cardiothoracic surgery and surgical closure was recommended. She underwent repair of the sinus of Valsalva aneurysm with a Dacron graft, closure of the aorto-right ventricular fistula with a CorMatrix patch, reimplantation of the right coronary artery, ligation of the circumflex artery-PA fistula, and tricuspid valve repair with an Edwards annuloplasty ring. The patient had an unremarkable postop course and was discharged home.

## 3. Discussion

Fistulas of the coronary sinus are rare. The aortic right ventricular fistula falls within abnormal vascular connections of the aorta named aortocameral fistulas. The diagnosis is usually made by transthoracic or transesophageal echocardiography, cardiac catheterization with aortography, or MRI. Congenital sinus of Valsalva aneurysms is usually undetected until they rupture, which usually occurs in the third or fourth decade of life [5]. The risk of rupture is about 0.4% [6]. Closure of an aortic right ventricular fistula in asymptomatic patients is recommended due to the low rate of procedure related complications and the risk of heart failure, bacterial endocarditis, pulmonary vascular disease, aneurysm formation, and spontaneous rupture. Treatment options include surgery and, more recently, percutaneous closure; however the optimal management remains unclear. On the other hand, coronary artery fistulas are the most common hemodynamically significant congenital coronary abnormality. They account for 0.2–0.4% of all congenital cardiac anomalies. This anomaly may lead to heart failure, infectious endocarditis, and arrhythmia. Spontaneous closure is rare and surgical closure is usually required [7]. The patient, therefore, required and underwent surgical closure.
